# Shigellosis and Cryptosporidiosis, Baltimore, Maryland

**DOI:** 10.3201/eid1207.060449

**Published:** 2006-07

**Authors:** David M. Hartley, Karl C. Klontz, Patricia Ryan, J. Glenn Morris

**Affiliations:** * University of Maryland School of Medicine, Baltimore, Maryland, USA;; † The George Washington University School of Public Health and Health Services, Washington, DC, USA;; ‡ US Food and Drug Administration, College Park, Maryland, USA;; § Maryland Department of Health and Mental Hygiene, Baltimore, Maryland, USA

**Keywords:** weather, climate, disease outbreaks, letter

**To the Editor:** Floret et al. argue convincingly that natural disasters, including severe floods and windstorms, tend not to result in epidemics of infectious disease (*1*). This conclusion is consistent with the lack of epidemics of shigellosis and cryptosporidiosis after hurricane rains in Baltimore, Maryland.

Shigellosis and cryptosporidiosis are associated with waterborne and foodborne transmission (*2**,**3*). We examined Baltimore shigellosis and cryptosporidiosis incidence to assess whether disease risk was related to temperature or rainfall from January 1, 1998, to December 31, 2004. Maryland FoodNet supplied case data; population estimates were acquired from the Maryland Department of Planning State Data Center; and meteorologic data for Baltimore Washington International airport (≈10 miles from the city center) were obtained from the National Atmospheric and Oceanic Administration (*4*).

During the study period, 38 cases of cryptosporidiosis and 943 cases of shigellosis were reported in Baltimore. Temperature was strongly seasonal; precipitation was not. A dry period during 1999 was observed. No seasonal cryptosporidiosis patterns were identifiable. Two outbreaks of shigellosis occurred; in 2000 (≈50 cases) and 2002–2004 (≈870 cases). Sporadic cases of shigellosis were not seasonal.

Two hurricanes resulted in heavy rainfall in Baltimore during the study period (*5*). Hurricane Floyd inundated the city with rain on September 16, 1999, and on September 19, 2003, Hurricane Isabel produced heavy rains and storm surge in Baltimore (which is located near the northern end of Chesapeake Bay). Approximately 4 other named tropical storms or depressions directly affected Baltimore rainfall during the study. However, collectively, none of these events had distinguishable signatures in the incidence of shigellosis or cryptosporidiosis in this urban environment.

The institutional review boards of the University of Maryland School of Medicine, The George Washington University Medical Center, and the Maryland Department of Health and Mental Hygiene approved this study. Dr Hartley is supported by a National Institutes of Health Career Development Award (K25 AI-58956).
